# Particular Burns

**Published:** 1907-09

**Authors:** E. C. Clark

**Affiliations:** Chapman, Ga.


### PARTICULAR BURNS.*

By E. C. Clark, M. D., Chapman, Ga.

---

Mr. President and Gentlemen:

Before presenting this simple epistle I wish to read not exactly a preface, nor is it as the boy said, "a front piece," but a preface. When I received the invitation from our faithful secretary to attend and bring with me a paper and read on this occasion, I first decided that to do this, I would greatly embarrass an

*Read before the Ocmulgee Medical Association, Eastman, Ga., July 16, 1907-

honorable, experienced, intelligent, audience. And, this I could not afford under any circumstances to do. And, that I might be like the soldier assigned to foreign duty who said; "I'll tell you, boys, I am scared, and I am bad scared; I do wish I was a baby, and how I do wish I was a gal baby." Though I told a party to tell him, (Dr. Miller) that I would try and attend and bring with me an up-to-date news-paper and read there-from some selected paragraph. Thinking more over the matter of my inexperience on this line, that duty in time nurtured progress, and progress well cultivated availed success, all depending upon prompt response to opportuned duty, a few lines on which I quote;

They do me wrong who say I come no more,
  When once I knock and fail to find you in,
For every day I stand out-side your door,
  And bid you wake, to fight and win.

Wail not for precious chances passed a way,
  Weep not for golden chances on the wane.
Each night I burn the records of the day,
  At sunrise every soul is born again.

Laugh like the boy at splendors that have sped.
  To vanquished joys be blind and deaf and dumb,
My Judgment seals the dead past with its dead.
  But never binds a moment yet to come.
  I here-to attach a few run down thoughts.

A PARTICULAR BURN.

Its Extent, Treatment, Duration,, and Results.

One afternooon late in the fall of 1905 I received a hasty call to see Miss W. 13 years of age, robust and healthy, who from having her clothes burned, was completely cered from axilla line including her right arm, down to upper 1-3 of legs, and from axilla line up and over the clavicle and scapular region to angry blister burn. She was suffering very little from cered portion, but intensely from blistered portion. Considering everything as presented though there was nothing necessary to be done but to relieve pain, I gave her morphine 1-4 of a grain waited 1-2 hr. without results, repeated dose, resulting in about an hour's sleep. Waking up I noticed her courageous hold up as I proceeded to dress her with "Unguentine" and prescribed morphia sufficiently to control severe pain, salts to keep bowels open. Left her thinking it would be but a few hours when all would be over. Hearing nothing more until the third day I was called back saying she was yet alive. Arriving, I found her resting very well, temperature 101 deg, pulse 90, and a very good volume. Removing the original dressing I found the cered portion had changed into a general mass of sloughs. Not a particle of epidermis, and but little skin proper could be seen intact. These facts before me, situated at some distance in the country, in a half sheltered little log cabin, not a crack ceiled, the cold winds puffing in and through the house, not a single person present sufficiently reliable to nurse her, nothing suitable for nourishment, and but little possibility of getting it, being one of the poorest of the poor presented to me the most forsaken, pathetic, horrible scene ever witnessed in my humble experience. Redressing her with the old remedy, caron's oil, this being all at hand at this time, prescribed about the same routine treatment except a little quinine.

The next day was informed she was yet living and so far as they could tell was about the same. On the 7th day, was called again saying she had grown much worse. Arriving I found her very restless, still suffering but little from burned surface, but intensely in the region of the appendix. Examination revealed a complete perforation of the abdominal wall apparently from external sloughing. Pulse 140, temp. 104 deg., tongue slightly coated and kidneys very inactive. No visible sign of internal trouble and practically no chance of making the usual examination for appendicitis without anesthesia and thinking certainly it would be but a short time when death would end it, I followed about the same treatment except a little strychnine added. On the 9th day was informed she had died that afternoon, falling into a stupor several hours before, and passed very quietly away. No part of the cered portion ever appeared other than intensely anaemic except about an inch around this said perforation, which was very angry. This patient survived according to general rules, an excess of at least 1-3 surface, and about 8 1-2 days time. The information, if you will pardon me, I will ask as to what was due her living so long, why did she suffer so little from the extensive burn? Why did parts remain so anaemic? Was the perforation as result of the burn direct, or was it appendicitis, if appendicitis, was it the result of the burn, or was it traumatic?

